# Urogenital symptoms in mitochondrial disease: overlooked and undertreated

**DOI:** 10.1111/ene.13952

**Published:** 2019-04-30

**Authors:** O. V. Poole, T. Uchiyama, I. Skorupinska, M. Skorupinska, L. Germain, D. Kozyra, S. Holmes, N. James, E. Bugiardini, C. Woodward, R. Quinlivan, A. Emmanuel, M. G. Hanna, J. N. Panicker, R. D. S. Pitceathly

**Affiliations:** ^1^ MRC Centre for Neuromuscular Diseases UCL Queen Square Institute of Neurology and National Hospital for Neurology and Neurosurgery London UK; ^2^ Department of Uro‐Neurology UCL Queen Square Institute of Neurology and National Hospital for Neurology and Neurosurgery London UK; ^3^ Department of Neurology School of Medicine International University of Health and Welfare Chiba Japan; ^4^ Department of Neurology School of Medicine International University of Health and Welfare Ichikawa Hospital Chiba Japan; ^5^ Neurogenetics Unit National Hospital for Neurology and Neurosurgery London UK; ^6^ Dubowitz Neuromuscular Centre Great Ormond Street Hospital London UK; ^7^ Gastro‐Intestinal Physiology Unit University College London Hospital London UK

**Keywords:** bladder symptoms, bowel symptoms, lower gastrointestinal tract symptoms, lower urinary tract symptoms, mitochondrial disease, sexual dysfunction, urogenital symptoms

## Abstract

**Background and purpose:**

Bowel symptoms are well documented in mitochondrial disease. However, data concerning other pelvic organs is limited. A large case–control study has therefore been undertaken to determine the presence of lower urinary tract symptoms (LUTS) and sexual dysfunction in adults with genetically confirmed mitochondrial disease.

**Methods:**

Adults with genetically confirmed mitochondrial disease and control subjects were recruited from a specialist mitochondrial clinic. The presence and severity of LUTS and their impact on quality of life, in addition to sexual dysfunction and bowel symptoms, were captured using four validated questionnaires. Subgroup analysis was undertaken in patients harbouring the m.3243A>G *MT‐TL1* mitochondrial DNA mutation. A subset of patients underwent urodynamic studies to further characterize their LUTS.

**Results:**

Data from 58 patients and 19 controls (gender and age matched) were collected. Adults with mitochondrial disease had significantly more overactive bladder (81.5% vs. 56.3%, *P* = 0.039) and low stream (34.5% vs. 5.3%, *P* = 0.013) urinary symptoms than controls. Urodynamic studies in 10 patients confirmed that bladder storage symptoms predominate. Despite high rates of LUTS, none of the patient group was receiving treatment. Female patients and those harbouring the m.3243A>G *MT‐TL1* mutation experienced significantly more sexual dysfunction than controls (53.1% vs. 11.1%, *P* = 0.026, and 66.7% vs. 26.3%, *P* = 0.011, respectively).

**Conclusions:**

Lower urinary tract symptoms are common but undertreated in adult mitochondrial disease, and female patients and those harbouring the m.3243A>G *MT‐TL1* mutation experience sexual dysfunction. Given their impact on quality of life, screening for and treating LUTS and sexual dysfunction in adults with mitochondrial disease are strongly recommended.

## Introduction

Mitochondrial disorders are genetic diseases caused by mutations in nuclear encoded or mitochondrial DNA (mtDNA) encoded genes that impair oxidative phosphorylation, with resultant reduced ATP generation. The adult prevalence is approximately 1 in 4300 1, ranking mitochondrial diseases amongst the most commonly inherited neurological disorders. Central neurological and neuromuscular phenotypes predominate in adults given the high energy requirements of the nervous system. Gastrointestinal (GI) symptoms are common 2 and are cardinal features of mitochondrial neurogastrointestinal encephalomyopathy 3 and an important manifestation of the m.3243A>G *MT‐TL1* mtDNA mutation 4. Given that mitochondrial GI symptoms are likely to be underpinned by impaired oxidative phosphorylation within the smooth musculature or autonomic nervous system, there is a high probability that the genitourinary tract is impaired by similar pathophysiological mechanisms. However, systematic evaluation of lower urinary tract symptoms (LUTS) and sexual dysfunction has not been undertaken in genetically confirmed mitochondrial disease.

A large case–control study has therefore been undertaken to determine the frequency, profile and potential pathophysiological mechanisms underpinning LUTS and sexual dysfunction in adults with genetically confirmed mitochondrial disease.

## Methods

### Study population

Adults (aged ≥ 18 years) with genetically confirmed mitochondrial disease attending our specialist mitochondrial disorders clinic, alongside age and gender matched control subjects (partners, unaffected relatives or friends of patients), were recruited. Demographic and clinical data, including genotype, time from diagnosis and medications, were collected. For patients harbouring the m.3243A>G *MT‐TL1* mutation blood heteroplasmy level and the presence/absence of diabetes mellitus (DM) were recorded. The Newcastle Mitochondrial Disease Adult Scale (NMDAS) score 5 was completed as part of routine clinical assessments. The study was approved by the Queen Square Research Ethics Committee, London (09/H0716/76), and informed consent was obtained.

### Assessment of lower urinary tract symptoms, sexual dysfunction and bowel symptoms

Lower urinary tract symptoms, sexual dysfunction and bowel symptoms were evaluated using four validated, self‐administered questionnaires: (i) the Urinary Symptom Profile (USP) 6; (ii) the SF‐Qualiveen (SFQ) 7; (iii) the Arizona Sexual Experiences Scale (ASEX) 8; and (iv) the Neurogenic Bowel Dysfunction (NBD) score 9.

The USP scores three symptom domains: stress urinary incontinence (SUI, 0–9); overactive bladder (OAB, 0–21); and low stream (LS, 0–9) (higher score indicating greater severity). The presence/absence of global LUTS (total USP score >0 or 0, respectively), domain LUTS (individual USP sub‐score >0 or 0, respectively) and symptom severity (sub‐score) for each of the three domains was analysed. The SFQ is validated for use in neurological patients and assesses LUTS‐related quality of life (QoL). A higher score indicates greater impact on QoL. The presence of any impact on QoL (score > 0) and its severity (total score) were analysed in those with symptoms detected by the USP. The ASEX evaluates sexual dysfunction. It comprises five questions, with the last two completed only by individuals sexually active within the last week. The presence or absence of recent sexual activity and sexual dysfunction were analysed. The NBD comprises 10 questions concerning bowel function. The presence/absence of symptoms (score ≥1 or 0 respectively) was analysed.

### Urodynamic studies

Urodynamic studies (UDS) were undertaken in a subset of patients with symptoms identified using the USP questionnaire, to further characterize the LUTS and guide management. These comprised non‐invasive uroflowmetry, measurement of post‐void residual (PVR) volumes and invasive multichannel UDS, including filling cytometry (medium fill at 50 ml/min) and pressure‐flow studies (Medical Measurement Systems, Dover, NH, USA) following International Continence Society Good Urodynamic Practices.

Data description and statistical analysis were performed according to data type (Data S1, Supporting Information).

## Results

### Demographics

Eighty‐eight genetically confirmed mitochondrial disease patients were offered two questionnaires, one for the patient and one to enable them to recruit an unaffected control subject. Questionnaires from 58 patients (mean age 46.2 ± 14.9 years, 67.2% female) and 19 age/sex matched control subjects (mean age 44 ± 13.5 years, 47.4% female; age *P* = 0.581, sex *P* = 0.121; Fig. S1, Tables 1 and S1) were completed. The average time from diagnosis was 9.6 ± 8.7 years. The m.3243A>G *MT‐TL1* mutation was the most common genotype in the patient cohort (26/58, 44.8%). Fifty‐three patients (91.4%) harboured pathogenic mutations in mtDNA.

**Table 1:** Demographic and molecular characteristics of the study population

	Patients		Controls		*P* value
	*n* (% total)	Age, years (SD)	*n* (% total)	Age, years (SD)	
All	58	46.2 (14.9)	19	44.0 (13.5)	Age: 0.581
Females	39 (67.2)	46.4 (15.3)	9 (47.4)	42.0 (13.3)	Age: 0.466
Males	19 (32.8)	45.9 (14.1)	10 (52.6)	45.6 (13.4)	Age: 0.959 Sex: 0.121
mtDNA genotype	53 (91.4)				
m.3243A>G	26 (44.8)	51.1 (14.2)	19	44.0 (13.5)	Age: 0.111
Single mtDNA deletion	12 (20.7)	41.8 (17.5)	19	44.0 (13.5)	Age: 0.705
m.8344A>G	5 (8.6)				
m.3260A>G	2 (3.4)				
m.9185T>C	2 (3.4)				
mtDNA duplication	2 (3.4)				
Other mtDNA mutation	4 (6.9)				
nDNA genotype	5 (8.6)				
*POLG*	2 (3.4)				
Other nDNA mutation	3 (5.2)				

mtDNA, mitochondrial DNA; nDNA, nuclear DNA.

### Lower urinary tract symptoms

**Figure 1:**
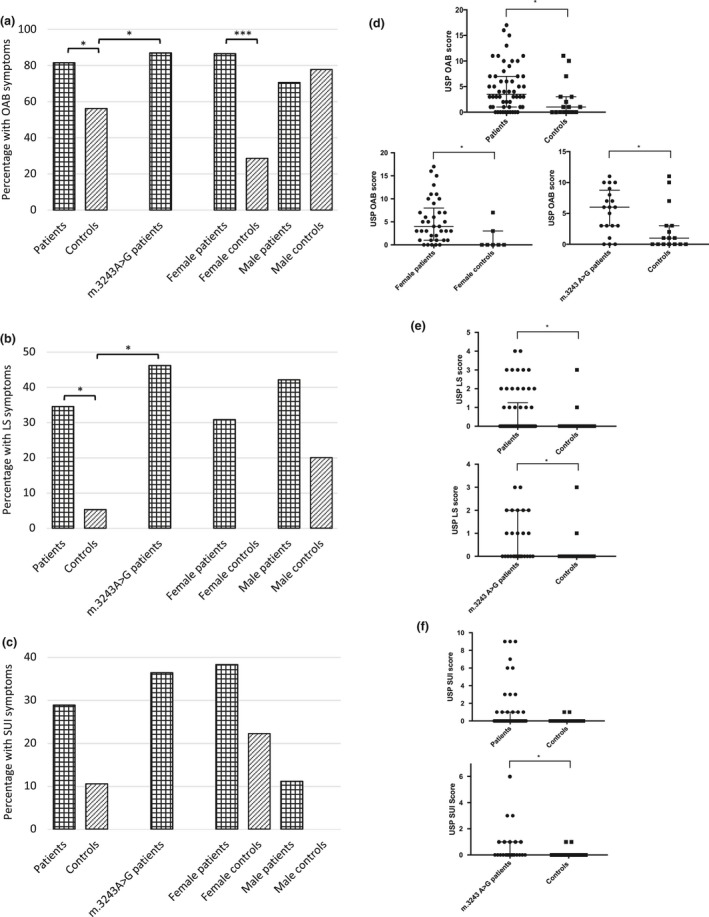
Urinary symptoms across domains of the Urinary Symptom Profile in adults with mitochondrial disease and controls. (a)–(c) Presence of symptoms in the total population and subgroups. (d)–(f) Severity of symptoms in the total population and subgroups demonstrating significant findings. LS, low stream; OAB, overactive bladder; SUI, stress urinary incontinence. *P < 0.05.

The USP detected LUTS in 41/49 (83.7%) patients compared with 11/16 (68.8%) controls (*P* = 0.030; Figs 1 and S2, Table 2). Forty‐four (44/54, 81.5%) patients experienced OAB symptoms, 20/58 (34.5%) LS symptoms and 15/52 (28.8%) SUI symptoms. Patients were significantly more likely to experience OAB (*P* = 0.039) and LS (*P* = 0.013) symptoms but not SUI symptoms (*P* = 0.109), with significantly higher scores (OAB *P* = 0.023 and LS *P* = 0.050) compared with controls. There was an impact on QoL from symptoms in 24/41 (58.5%) patients with LUTS, which was comparable with controls (Table S2). Female patients were more likely to have LUTS (29/33, 87.9% vs. 3/7, 42.9%, *P* = 0.007), particularly OAB symptoms (32/37, 86.5% vs. 2/7, 28.6%, *P* = 0.001), than female controls and their OAB symptoms were more severe (*P* = 0.013). No statistically significant differences were detected in the other USP domains or between male patients and controls.

**Table 2:** Presence and severity of urinary symptoms across Urinary Symptom Profile domains in adults with mitochondrial diseases and controls

USP domain	Patients					Controls					*P* value presence	*P* value severity
	Group	Completed domain, *n*	Symptoms present, *n* (%)	Severity of symptoms		Group	Completed domain, *n*	Symptoms present, *n* (%)	Severity of symptoms			
				Mdn	IQR				Mdn	IQR		
All domains	All	49	41 (83.7)			All	16	11 (68.8)			0.030*	
	Females	33	29 (87.9)			Females	7	3 (42.9)			0.007**	
	Males	16	12 (75.0)			Males	9	8 (88.9)			0.405	
	m.3243A>G	20	18 (90.0)			All	16	11 (68.8)			0.109	
OAB	All	54	44 (81.5)	3.5	6	All	16	9 (56.3)	1	3	0.039*	0.023*
	Females	37	32 (86.5)	4	6	Females	7	2 (28.6)	0	1.5	0.001***	0.013*
	Males	17	12 (70.6)	3	6	Males	9	7 (77.8)	1	2	0.694	0.659
	m.3243A>G	23	20 (87.0)	6	5.25	All	16	9 (56.3)	1	3	0.031*	0.018*
LS	All	58	20 (34.5)	0	1	All	19	1 (5.3)	0	0	0.013*	0.050*
	Females	39	12 (30.8)	0	1	Females	9	0 (0.0)	0	0	0.055	0.089
	Males	19	8 (42.1)	0	1.5	Males	10	2 (20.0)	0	0	0.234	0.322
	m.3243A>G	26	12 (46.2)	0	1.75	All	19	1 (5.3)	0	0	0.003**	0.016*
SUI	All	52	15 (28.8)	0	1	All	19	2 (10.5)	0	0	0.109	0.079
	Females	34	13 (38.2)	0	1	Females	9	2 (22.2)	0	0	0.370	0.272
	Males	18	2 (11.1)	0	0	Males	10	0 (0.0)	0	0	0.274	0.524
	m.3243A>G	22	8 (36.4)	0	1	All	19	2 (10.5)	0	0	0.055	0.049*

IQR, interquartile range; LS, low stream; Mdn, median; *n*, number; OAB, overactive bladder; SUI, stress urinary incontinence. Presence of symptoms indicated by Urinary Symptom Profile (USP) score in each domain >0; severity of symptoms as indicated by total USP score in each domain. *P < 0.05; **P < 0.01; ***P < 0.001, at four decimal places.

### Urodynamic studies

Uroflowmetry (*n* = 10) showed an abnormal flow pattern in eight patients. Mean maximum flow rate (*Q*_max_) was 18.3 ml/s (range 4.3–36.7; reduced flow rate < 15 ml/s, *n* = 6). Mean PVR was 49.0 ml (range 0–345 ml). Six patients underwent invasive UDS. Abnormal findings in filling cystometry included detrusor overactivity (*n* = 2), increased bladder sensations (*n* = 3; detrusor overactivity in 2), reduced bladder sensations (*n* = 3), and increased total bladder capacity (*n* = 3). A pressure‐flow study showed abnormalities (*n* = 4) and none showed an obstructed flow (Table 3).

**Table 3:** Uroflowmetry and invasive urodynamics in adults with mitochondrial disease

Gender	Age (years)	Confirmed mutation	Uroflowmetry				Urodynamics: filling cystometry					Urodynamics: pressure flow study				
			Voided volume (ml)	Pattern	*Q*_max_ (ml/s)	PVR (ml)	FSV (ml)	NDV (ml)	DO	Compliance	Total bladder capacity (ml)	Voided volume (ml)	Pattern	*Q*_max_ (ml/s)	Pdet at *Q*_max_ (cm H_2_O)	PVR (ml)
Female	42	m.3302A>G	305	Irregular	31.6	0	362↑	467	−	N	650↑	650	Irregular	32	27	0
Male	64	Multiple mtDNA deletions	272	Prolonged	4.3↓	345↑	574↑	637↑	−	N	722↑	22	Strain	2↓	0↓	700↑
Male	78	m.3243A>G	195	Irregular	14.6↓	0	235↑	451	−	N	499	586	Intermittent	7.8↓	22	0
Female	40	m.3243A>G	180	Irregular	12↓	0	61↓	122↓	+	N	83↓	†				
Female	58	m.3260A>G	145	Smooth	10↓	40	104↓	105↓	+	N	194	†				
Female	62	m.14674T>C	342	Intermittent	36.7	16	62↓	182↓	−	N	601↑	‡				
Female	59	Single deletion	222	Smooth	23.4	0										
Male	26	m.9185T>C	300	Intermittent	31.1	0										
Male	32	m.9185T>C	188	Intermittent	11.2↓	47										
Male	52	m.3243A>G	115	Irregular	7.6↓	42										

DO, detrusor overactivity; FSV, bladder volume at first sensation; N, normal; NDV, bladder volume at normal desire to void; Pdet, maximum detrusor pressure; *Q*_max_, maximum flow rate; +, positive; −, negative; †, not recorded because of severe DO; ‡, patient unable to void; ↓, decreased below the reference range; ↑, increased above the reference range.

### Sexual dysfunction

**Figure 2:**
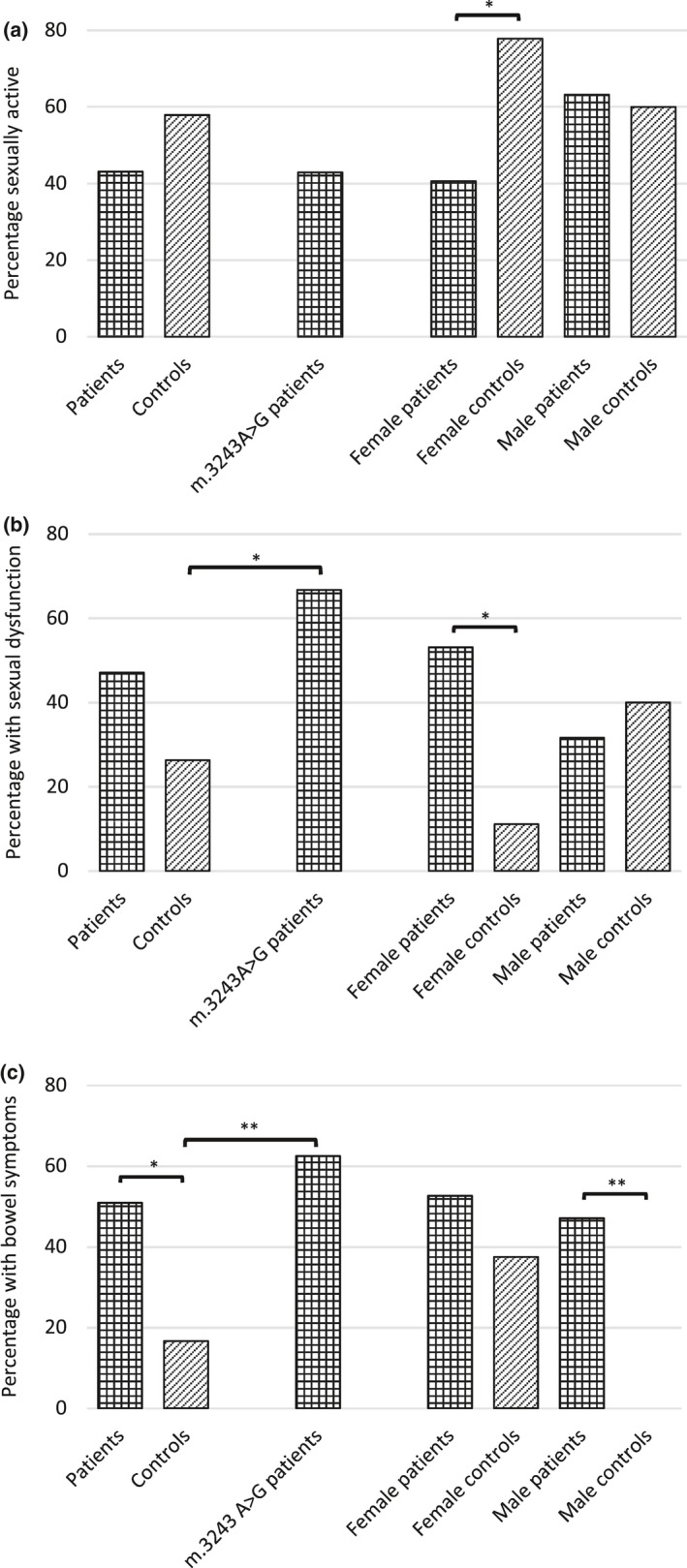
Sexual activity (a), sexual dysfunction (b) and bowel symptoms (c) in adults with mitochondrial disease and controls. *P < 0.05, **P < 0.01.

Fifty‐one patients completed the ASEX questionnaire. Twenty‐two (43.1%) were sexually active within the previous week and 24 (47.1%) experienced sexual dysfunction. No differences in sexual activity or the presence of sexual dysfunction were detected between patients and controls (*P* = 0.271 and *P* = 0.100, respectively). Fewer female patients than controls had been sexually active (13/32, 40.6% vs. 7/9, 77.8%, *P* = 0.049) and more experienced sexual dysfunction (17/32, 53.1% vs. 1/9, 11.1%, *P* = 0.026). No difference was observed between rates of sexual activity or sexual dysfunction in males compared with controls (Fig. 2, Table 4).

**Table 4:** Sexual activity, sexual dysfunction and bowel symptoms in adults with mitochondrial disease and controls

Patient group				Control group				Sexually active *P* value	Sexual dysfunction *P* value	Bowel symptoms *P* value
	Sexually active *n*/total *n* (%)	Sexual dysfunction *n*/total *n* (%)	Bowel symptoms *n*/total *n* (%)		Sexually active *n*/total *n* (%)	Sexual dysfunction *n*/total *n* (%)	Bowel symptoms *n*/total *n* (%)			
All	22/51 (43.1)	24/51 (47.1)	28/55 (50.9)	All	11/19 (57.9)	5/19 (26.3)	3/18 (16.7)	0.271	0.100	0.011*
Females	13/32 (40.6)	17/32 (53.1)	20/38 (52.6)	Females	7/9 (77.8)	1/9 (11.1)	3/8 (37.5)	0.049*	0.026*	0.437
Males	12/19 (63.2)	6/19 (31.6)	8/17 (47.1)	Males	6/10 (60.0)	4/10 (40.0)	0/10 (0.0)	0.868	0.650	0.010**
m.3243A>G	9/21 (42.9)	14/21 (66.7)	15/24 (62.5)	All	11/19 (57.9)	5/19 (26.3)	3/18 (16.7)	0.342	0.011*	0.003**

*n*, number. Sexual activity within the last week and sexual dysfunction as indicated by the Arizona Sexual Experiences Scale. Bowel symptoms as indicated by a Neurogenic Bowel Dysfunction score >0. *P < 0.05; **P < 0.01, at four decimal places.

### Bowel symptoms

Significantly more patients had bowel symptoms than controls (28/55, 50.9% vs. 3/18, 16.7%, *P* = 0.011; Fig. 2, Table 4) and 29.8% (17/57) of patients reported bowel opening two to six times a week or less, compared with 3.5% (2/57) who experienced faecal incontinence.

### Relationship between lower urinary tract symptoms, sexual dysfunction and bowel symptoms

Bowel symptoms and sexual dysfunction coexisted in patients (*P* = 0.011), but there was no association between LUTS (USP global or domain scores) and sexual or bowel dysfunction.

### Disease burden and lower urinary tract symptoms, sexual dysfunction and bowel symptoms

The scaled NMDAS score (*n* = 56) was significantly higher in patients with LUTS compared to without (median 16.7 vs. 7.3, *P* = 0.033). There was a significant correlation between NMDAS and USP LS scores (*r* = 0.296, *P* = 0.027). However, no correlation was detected with the USP SUI or OAB sub‐scores (Fig. S3) and there was no difference in NMDAS score in patients with and without sexual (*P* = 0.080) or bowel dysfunction (*P* = 0.364).

### Treatments for lower urinary tract symptoms, sexual dysfunction and bowel symptoms

Compared to controls, no patients were receiving treatment for LUTS (controls 2/19, 10.5% vs. patients 0/58, 0.0%, *P* = 0.012). The proportions of patients and controls on treatment for bowel (5/58, 8.6% vs. 0/19, 0.0%, *P* = 0.186) or sexual dysfunction symptoms (4/58, 6.9% vs. 2/19, 10.5%, *P* = 0.609) were comparable (Table S3).

### Genotype‐specific findings: the m.3243A>G *MT‐TL1* mutation

Patients harbouring the m.3243A>G *MT‐TL1* mutation (*n* = 26) had more OAB (*P* = 0.031) and LS (*P* = 0.003) symptoms than controls, and scored higher in all three USP domains (OAB *P* = 0.018, LS *P* = 0.016, SUI *P* = 0.049; Fig. 1, Table 2) although, on removal of the outlier, significance in the SUI domain was lost (*P* = 0.103). Sexual activity was comparable with controls (*P* = 0.342), but patients were more likely to experience sexual dysfunction (14/21, 66.7% vs. 5/19, 26.3%, *P* = 0.011). They were also more likely to have bowel symptoms (15/24, 62.5% vs. 3/18, 16.7%, *P* = 0.003; Fig. 2, Table 4).

Blood heteroplasmy levels were available for 17/26 patients with the m.3243A>G *MT‐TL1* mutation. There was no significant positive correlation between blood heteroplasmy levels and domains of the USP (Fig. S4). There was also no difference between heteroplasmy levels in those with or without sexual dysfunction (*P* = 0.325) or bowel symptoms (*P* = 0.101).

Eighteen patients with the m.3243A>G *MT‐TL1* mutation had DM. There was no association between the presence of DM and LUTS (*P* = 0.515) and no difference between scores in individual domains of the USP in patients with and without DM (Fig. S5). Sexual dysfunction (*P* = 0.655) and bowel symptoms (*P* = 0.132) were not associated with DM.

## Discussion

Lower urinary tract symptoms are increasingly reported in inherited neurological disorders that involve the central and peripheral nervous systems 10, 11. However, limited data on LUTS in mitochondrial diseases exist 12, 13. The results of this study suggest that adults with genetically proven mitochondrial disease frequently experience LUTS (83.7%). In our cohort, OAB (storage) symptoms were the most common (81.5%), followed by LS (voiding) symptoms (34.5%) and SUI symptoms (28.8%), and OAB and LS symptoms were significantly more common in patients than controls. Sexual dysfunction was associated with mitochondrial disease in female patients and carriers of the m.3243A>G *MT‐TL1* mutation (53.1% and 66.7% respectively). Bowel symptoms were more common in patients (50.9%) and were associated with sexual dysfunction.

The results of urodynamic testing in a representative number of patients from the cohort suggests that the lower urinary tract is affected in numerous ways in mitochondrial disorders, including effects on storage and voiding sensory and motor functions. Storage dysfunction was due to detrusor overactivity and increased bladder sensations. In neurological disorders, storage dysfunction occurs most frequently with suprasacral lesions 14 and could be related to a central neurological phenotype in patients with mitochondrial disease, e.g. mitochondrial encephalomyopathy, lactic acidosis and stroke‐like episodes (MELAS). Detrusor overactivity has also been reported in other primary muscle disorders such as the dystrophinopathies 11. However, it remains uncertain whether detrusor overactivity in these disorders is associated with the myopathic presentation or additional central nervous system or autonomic involvement. Although both sexes experienced comparable levels of OAB symptoms, only females reported significantly more OAB symptoms, and of greater severity, compared to age/gender matched controls. This discrepancy may result from the high rates of storage symptoms in the male control population (Fig. 1) due to other causes, such as benign prostatic enlargement which is present in half of men over 50 15. Sixty‐two questionnaires provided to control subjects were not returned, the explanation for which is probably multi‐factorial; for instance, the questionnaire might not have been passed on to the control subject by the patient or may not have been completed due to its sensitive nature or the absence of symptoms.

Voiding dysfunction, evidenced by an abnormal flow and/or elevated post‐void residual or detrusor underactivity in the pressure‐flow study, was observed in most patients. In neurological diseases this may arise from detrusor sphincter dyssynergia or an underactive detrusor 14. In our cohort, detrusor underactivity is likely to be an important cause based on the findings from four of the pressure‐flow studies. The cause for detrusor underactivity is unclear and could be due to involvement of the detrusor smooth muscle, the innervation of the lower urinary tract or diminished cellular energy production. Structural urological lesions such as benign prostatic enlargement cause voiding symptoms 14. However, the presence of symptoms in the all‐patient group, and lack of evidence for outflow obstruction in the patients undergoing urodynamic testing, implicates mitochondrial dysfunction. The apparent mismatch between the number of patients with voiding symptoms detected by the USP questionnaire and the high prevalence of voiding dysfunction in urodynamic testing (six of 10 cases) may be due to selection bias, given that only patients with significant LUTS agreed to undergo urodynamic testing. However, this also raises the possibility of asymptomatic voiding dysfunction.

Male sexual dysfunction has previously been evaluated in a questionnaire‐based screening study of autonomic function in MELAS and no significant difference with controls was detected 12. In our cohort, fewer female patients were sexually active than controls, and females and carriers of the m.3243A>G *MT‐TL1* mutation experienced significantly more sexual dysfunction. However, the confounding effect of a chronic disease cannot be completely excluded 16.

Despite the high prevalence of LUTS and their impact on QoL, no patients were on treatment for these symptoms. Further studies are required to explore LUTS and sexual dysfunction in mitochondrial disease and the reasons underpinning the treatment gap observed. These are likely to be multi‐factorial, including a lack of awareness amongst healthcare professionals concerning LUTS in mitochondrial disease and reluctance for patients to discuss them. Given that several safe and effective treatments exist for LUTS in patients with neurological disease 14, in addition to potential management options for sexual dysfunction, it is important to establish the presence of these symptoms. However, caution is advised when commencing treatments for lower urinary tract storage symptoms, particularly antimuscarinic agents which are associated with an increased risk of developing voiding dysfunction and incomplete bladder emptying when subclinical voiding problems exist. It is recommended that the established investigative approach is followed for neurological patients 14.

Fifty‐one per cent of our patient cohort, and 62.5% of m.3243A>G patients, had bowel symptoms, consistent with previous studies 2, 12. Male patients had significantly more bowel symptoms than same sex controls, a difference not seen in females. This may reflect the higher incidence of GI symptoms in females in the general population 17. Patients experienced more voiding difficulties (infrequent bowel opening, 29.8%) than faecal incontinence (3.5%) mirroring the higher prevalence of urinary voiding symptoms compared with SUI urinary symptoms.

Mitochondrial DM occurs in up to 42% of patients harbouring the m.3243A>G *MT‐TL1* mutation 18 and dysautonomia associated with DM is a well‐recognized cause of urinary tract, sexual and GI symptoms 19. However, no significant difference in these symptoms was seen in the m.3243A>G patients with or without DM, suggesting that their symptoms relate directly to their mitochondrial disorder rather than diabetes, in keeping with data investigating the influence of diabetes on the GI manifestations of m.3243A>G‐related mitochondrial disease 20.

Finally, total NMDAS scores correlated with LS but not OAB symptoms, whilst blood m.3243A>G heteroplasmy levels demonstrated no significant positive association with LUTS, sexual dysfunction or bowel symptoms. These findings might imply that disease burden does not influence the emergence of dysfunction, but might also reflect the challenges of using the NMDAS, originally designed to record longitudinal natural history data in individuals with mitochondrial disease, when comparing patients with heterogeneous clinical phenotypes 5. The results also emphasize the limited value of heteroplasmy levels, in particular when undertaken in blood rather than urinary epithelial cells or skeletal muscle tissue, when measuring disease severity 21.

In conclusion, the first cohort study of LUTS and sexual dysfunction in adults with mitochondrial disease is reported. It is confirmed that LUTS are common and impact QoL, and that female patients and those with the m.3243A>G *MT‐TL1* mutation experience sexual dysfunction. Bladder storage symptoms predominate, although voiding dysfunction may occur subclinically. Despite several effective treatments for LUTS in neurological diseases, none of the patients was receiving therapy for these, thus emphasizing the importance of active screening, investigation and appropriate interventions to improve management of adults with mitochondrial disease.

## Disclosure of conflicts of interest

Tomoyuki Uchiyama: International Continence Society/Pfizer International Fellowship 2017. All the other authors declare no financial or other conflicts of interest.

## Supporting information


**Data S1.** Data analysis.
**Figure S1.** Flow chart of patient and control recruitment.
**Figure S2.** Severity of urinary symptoms across USP domains in adults with mitochondrial disease and controls in subgroups without significant findings.
**Figure S3.** Correlation between the Newcastle Mitochondrial Disease Adult Scale and domains of the USP.
**Figure S4.** Correlation between m.3243A>G blood heteroplasmy levels and scores in the domains of the USP.
**Figure S5**. USP domains for patients harbouring the m.3243A>G mutation with and without diabetes.
**Table S1.** Comparison of age, sex and NMDAS scores between eligible adults with mitochondrial disease who completed and returned the questionnaire and those who did not
**Table S2**. Presence and severity of impact on quality of life from urinary symptoms in adults with mitochondrial disease and urinary symptoms
**Table S3.** Percentage of participants currently receiving treatment for symptoms of pelvic organ dysfunction
