# Supplementary material for: Urogenital symptoms in mitochondrial disease: overlooked and undertreated
Source: Eur J Neurol. 2019 Apr 30;26(8):1111–20. doi: 10.1111/ene.13952 (PMC6767393; doi:10.1111/ene.13952)
Supplement: Supplementary file 1 — Data S1. Data analysis. Figure S1. Flow chart of patient and control recruitment. Figure S2. Severity of urinary symptoms across USP domains in adults with mitochondrial disease and controls in subgroups without significant findings. Figure S3. Correlation between the Newcastle Mitochondrial Disease Adult Scale and domains of the USP. Figure S4. Correlation between m.3243A>G blood heteroplasmy levels and scores in the domains of the USP. Figure S5. USP domains for patients harbouring the m.3243A>G mutation with and without diabetes. Table S1. Comparison of age, sex and NMDAS scores between eligible adults with mitochondrial disease who completed and returned the questionnaire and those who did not Table S2. Presence and severity of impact on quality of life from urinary symptoms in adults with mitochondrial disease and urinary symptoms Table S3. Percentage of participants currently receiving treatment for symptoms of pelvic organ dysfunction [file ENE-26-1111-s001.docx]

**SUPPLEMENTARY MATERIAL**

**Data S1. Data analysis**

Quantitative variables (e.g. age) were described with the mean and standard deviation. Severity of symptoms in each domain of the USP and total SFQ score were described using the median and interquartile range. Qualitative variables, such as the presence or absence of symptoms, were presented as percentages. Results were compared between all patients and controls, those of the same sex, and between patients in common genotype subgroups and controls. Statistical analysis of quantitative data was performed using the t-test, for parametric data, and the Mann-Whitney test, for non-parametric data. Qualitative data was analysed using Chi-square test. Correlations were investigated using Spearman Rank correlation. Statistical significance was defined as a p value <0.05. Statistical analysis was performed using GraphPad Prism (7.0d for Mac OS X, San Diego, California, USA).

**Figure S1:** Flow chart of patient and control recruitment

**
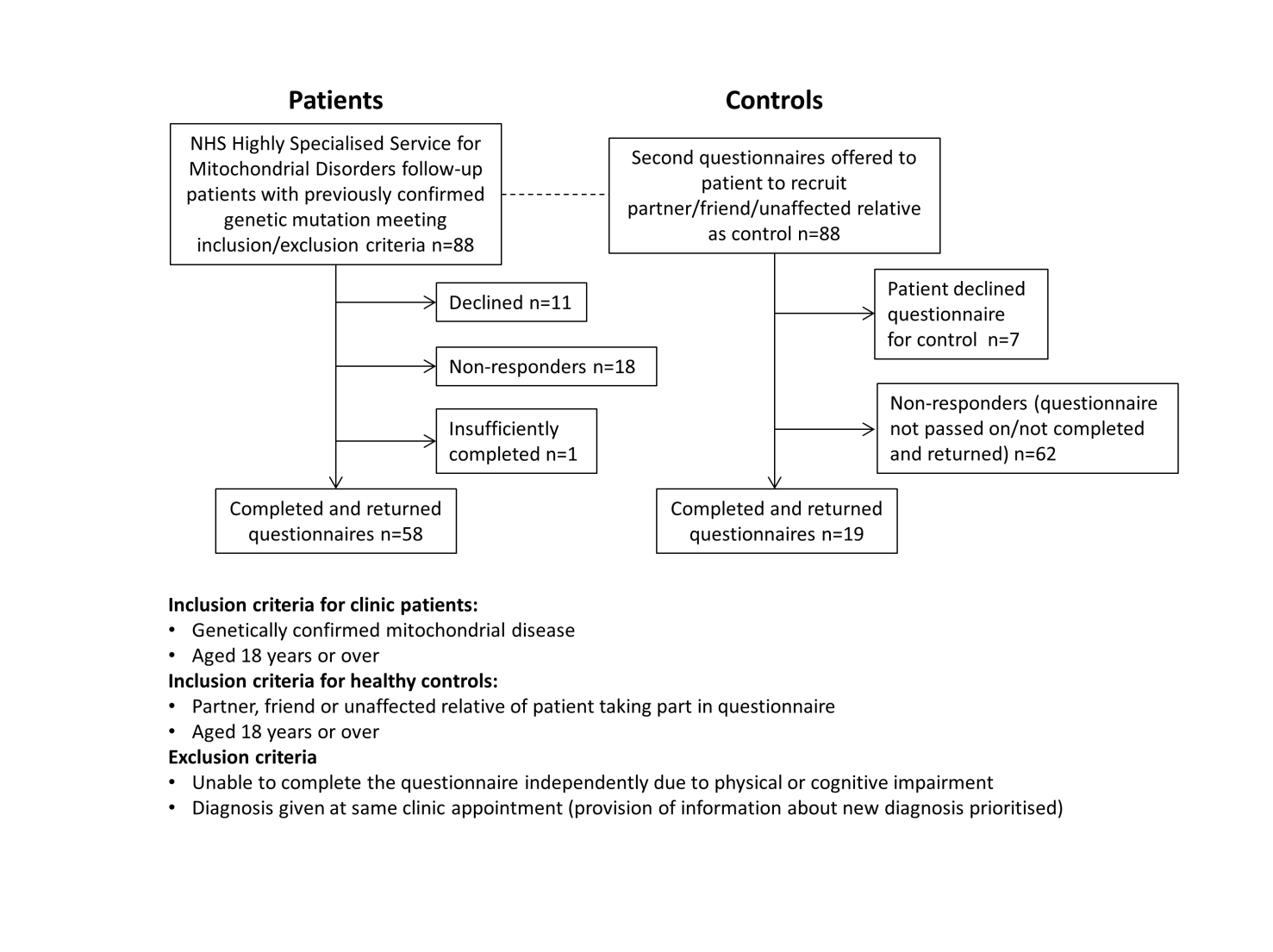
**

|  | Completed and returned questionnaires | | p value |
| --- | --- | --- | --- |
|  | **Yes** | **No** |  |
| Number (% total) | 58 (65.9) | 30 (34.1) |  |
| Age, yrs (SD) | 46.2 (14.9) | 45.8† (16.8) | 0.908 |
| Females (% total) | 39 (67.2) | 18† (62.1) | 0.632 |
| Median NMDAS score (n) | 15.8 (56) | 20.2† (28) | 0.498 |

**Table S1:** Comparison of age, sex and NMDAS scores between eligible adults with mitochondrial disease who completed and returned the questionnaire and those who did not

Abbreviations: SD, standard deviation. †Excluding insufficiently completed questionnaires.

**Figure S2:** Severity of urinary symptoms across USP domains in adults with mitochondrial disease and controls in subgroups without significant findings.

**Table S2**: Presence and severity of impact on quality of life from urinary symptoms in adults with mitochondrial disease and urinary symptoms

| **Patients** | | | | | **Controls** | | | | | **p value presence** | **p value severity** |
| --- | --- | --- | --- | --- | --- | --- | --- | --- | --- | --- | --- |
| **Group** | **Urinary symptoms detected by USP n** | **Presence of impact on QoL n (%)** | **Severity of symptoms (SFQ score)** | | **Group** | **Urinary symptoms detected by USP n** | **Presence of impact on QoL n (%)** | **Severity of symptoms (SFQ score)** | |  |  |
|  |  |  | **Mdn** | **IQR** |  |  |  | **Mdn** | **IQR** |  |  |
| **All** | 41 | 24 (58.5) | 0.25 | 1 | **All** | 11 | 5 (45.5) | 0 | 0.25 | 0.438 | 0.315 |
| **Females** | 29 | 17 (58.6) | 0.25 | 1 | **Females** | 3 | 2 (66.7) | 0.25 | 0.125 | 0.787 | 0.583 |
| **Males** | 12 | 7 (58.3) | 0.25 | 1.03125 | **Males** | 8 | 3 (37.5) | 0 | 0.375 | 0.361 | 0.509 |
| **m.3243A>G** | 18 | 11 (61.1) | 0.1875 | 0.46875 | **All** | 11 | 5 (45.5) | 0 | 0.25 | 0.411 | 0.507 |
| **SD** | 9 | 3 (33.3) | 0 | 1 | **All** | 11 | 5 (45.5) | 0 | 0.25 | 0.582 | 0.811 |

Abbreviations: IQR, inter-quartile range; Mdn, median; QoL, quality of life; SD, single mtDNA deletion

**Figure S3:** Correlation between Newcastle Mitochondrial Disease Scale for Adults and domains of USP

Abbreviations: LS, low stream; OAB, overactive bladder; SUI, stress urinary incontinence. *indicates p<0.05

**Table S3:** Percentage of participants currently receiving treatment for symptoms of pelvic organ dysfunction

|  | Patients on treatment | Percentage population | Controls on treatment | Percentage population | p value |
| --- | --- | --- | --- | --- | --- |
| Bladder treatment | 0/58 | 0.0% | 2/19 | 10.5% | 0.012* |
| Bowel treatment | 5/58 | 8.6% | 0/19 | 0.0% | 0.186 |
| Sexual dysfunction treatment | 4/58 | 6.9% | 2/19 | 10.5% | 0.609 |

*indicates p<0.05

**Figure S4:** Correlation between m.3243A>G blood heteroplasmy levels and scores in the domains of USP

Abbreviations: LS, low stream; OAB, overactive bladder; SUI, stress urinary incontinence.

**Figure S5**: USP domains for patients harbouring the m.3243A>G mutation with and without diabetes

Abbreviations: DM, diabetes mellitus; LS, low stream; OAB, overactive bladder; SUI, stress urinary incontinence.
